# Social Participation When Aging With an Early-Onset Neurological Disability: Protocol for Descriptive Qualitative Research

**DOI:** 10.2196/66963

**Published:** 2025-08-06

**Authors:** Mia Lapointe, Megan Veilleux, Pascale Simard, Hung Manh Nguyen, Angéline Labbé, Valérie Poulin, Samuel Turcotte

**Affiliations:** 1 School of Rehabilitation Sciences Faculty of Medicine Laval University Québec, QC Canada; 2 Department of Rehabilitation Interdisciplinary Research Center on Rehabilitation and Social Integration Québec, QC Canada; 3 Department of Occupational Therapy University of Quebec in Trois-Rivières Trois-Rivière, QC Canada

**Keywords:** social participation, active and healthy aging, gerontology, geriatrics, older adults, elder, elderly, older person, older people, aging, individuals aging with disabilities, neurological disability, traumatic brain injury, TBI, multiple sclerosis, MS, spinal cord injury, SCI, neurological illnesses, neurological diseases, neurological conditions, neuroscience, neuro, neurologists

## Abstract

**Background:**

Due to improvements in health care and rehabilitation, as well as better social conditions, individuals living with traumatic brain injury (TBI), multiple sclerosis (MS), or spinal cord injury (SCI) are living longer. It is therefore necessary to ensure the presence of social and health services adapted to the realities and specific needs of these populations aging with disabilities. Social participation is a key determinant of active aging and health. However, there is limited evidence regarding the social participation of these aging populations. To support the development of more inclusive approaches promoting the health of older adults, it is essential to better understand the diversity of social participation experiences among individuals aging with neurological disabilities.

**Objective:**

This study aims to explore how social participation is experienced by individuals aging with TBI, MS, or SCI; document the barriers and facilitators to their social participation; and explore avenues for interventions supporting their social participation.

**Methods:**

This descriptive qualitative research is part of a larger action research project conducted in partnership with individuals aging with disabilities, researchers, and community organizations providing services to these populations. Individuals 50 years or older living with TBI (n=8), MS (n=8), or SCI (n=8) will participate in a semistructured interview. The interviews will be transcribed verbatim, and the accuracy of the transcripts will be ensured through peer validation. Qualitative data will be analyzed using a mixed approach in alignment with the Framework methods. The use of the Human Development Model–Disability Creation Process (HDM-DCP) conceptual model will be used for deductive analysis. The coding tree will combine significant themes arising from the interviews’ inductive part and the themes from the HDM-DCP. Also, 12.5% of the analysis will be tested for stringency (ie, double-blind and interrater reliability exercise).

**Results:**

This study will provide insights into the diversity of social participation experiences of these populations as well as the influence of individual characteristics and environmental resources on their social participation.

**Conclusions:**

This project will lay the groundwork for the codevelopment of health promotion programs aimed at supporting the social participation of individuals aging with neurological disabilities. This study will also help to identify the resources and strengths that support social participation for these populations, as well as the systemic barriers that need to be addressed.

**International Registered Report Identifier (IRRID):**

DERR1-10.2196/66963

## Introduction

### Background

In Canada, one in four individuals has one or more disabilities [1]. Due to medical and rehabilitation advancements, as well as improved social conditions, individuals living with disabilities are experiencing a significant increase in life expectancy [2,3]. Consequently, the number of individuals aging with disabilities is on the rise [3], many of whom are impacted by long-term neurological conditions such as traumatic brain injury (TBI), multiple sclerosis (MS), and spinal cord injuries (SCI). Thus, these populations represent a significant proportion of individuals served in rehabilitation and community organizations, as they constitute a substantial percentage of neurological conditions in Canada [4,5].

Individuals aging with disabilities remain an understudied population [3,6-8], and there is limited evidence on the realities of aging with disabilities and the related needs. While research on aging with a disability is progressing, it remains marginal, particularly concerning the long-term experiences of individuals aging with early-onset neurological disabilities [9]. Available knowledge about the realities and social participation experiences of these populations is especially limited. There is a critical lack of representation of patient perspectives in the current literature. Addressing this gap is crucial for several reasons. First, sharing the perspectives of these individuals allows for a more comprehensive understanding of the challenges and opportunities they face, which can inform more effective and tailored interventions. Second, it empowers these individuals by giving them a voice in shaping policies and services that directly affect their lives. Finally, by highlighting their experiences, we can challenge societal misconceptions about aging with disabilities and promote a more inclusive approach to health care and social services. This hinders the development of appropriate solutions that support access to social and health services for individuals aging with disabilities and thus impedes the active aging of this population [10,11]. One such challenge is ensuring that individuals aging with disabilities have access to social and health services that are tailored to their specific needs and unique realities [12,13]. Continuity of access to social and health services adapted to the reality of individuals aging with disabilities is particularly important since obstacles to accessing social and health services, such as physical, cognitive, informational, and financial barriers, may lead to further restrictions in social participation [14]. To support this access, it is important to develop and improve solutions that enhance the availability, accessibility, acceptability, affordability, and usability of these services [15].

Furthermore, the aging of individuals with disabilities presents significant challenges, which compound the broader issues associated with the aging Canadian population [14,16]. The double stigma associated with ageism and disability can reduce the social participation of individuals aging with disabilities [17-21]. Levasseur et al [22] defined social participation as “a person’s involvement in activities providing interactions with others in community life and in important shared spaces, evolving according to available time and resources, and based on the societal context and what individuals want and is meaningful to them.” Although social participation is a key determinant of the health of aging populations [23], there is little scientific literature on the social participation of individuals aging with a neurological condition such as TBI, MS, or SCI [24].

This study is part of a larger research program aimed at developing health promotion programs to support the social participation of individuals aging with disabilities. In this descriptive exploratory qualitative study, the choice not to develop hypotheses is justified by several reasons. First, the exploratory nature of the research aims to examine a little-studied phenomenon without preconceived ideas, thus allowing an openness to unexpected discoveries. Second, the absence of predefined hypotheses helps to avoid potential biases that could influence the interpretation of the data and limit the open-mindedness necessary for in-depth exploration [25-27]. Finally, this approach aligns with the adopted socioconstructivist epistemological stance [28], which considers that knowledge is socially constructed through interactions, thus favoring the emergence of meaning from the data rather than the verification of pre-established hypotheses [25,26]. This study will contribute to laying the groundwork for the codevelopment of such programs by informing on how individuals aging with TBI, SCI, or MS would like to be supported in terms of social participation.

### Interest in Those Populations

Neurological disorders account for a significant number of health conditions in many countries worldwide [29,30]. In Canada, they have a substantial impact (ie, funding allocated to care and services, hospitalization rate) [4]. The choice of these 3 populations is based on the prevalence of MS in Canada, which is one of the highest in the world [31], and a high incidence of TBI in the country (approximately 456 individuals per day) [31,32]. Thus, by 2031, TBI will be among the most common neurological disorders in Canada [32]. The prevalence of SCI will rise to over 120,000 individuals, approximately more than 3600 new cases per year [33,34]. Finally, these populations offer a contrast between aging with a degenerative condition (MS) and nonprogressive conditions (TBI and SCI).

### Objectives

This study will pursue 3 objectives aimed at providing insights into the experiences and needs regarding the social participation of individuals aging with neurological disabilities. The objectives are as follows: 1) explore how social participation is experienced by individuals aging with TBI, MS, or SCI; 2) document the obstacles and facilitators to their social participation; 3) explore intervention strategies supporting their social participation.

## Methods

### Overview

**Figure 1:**
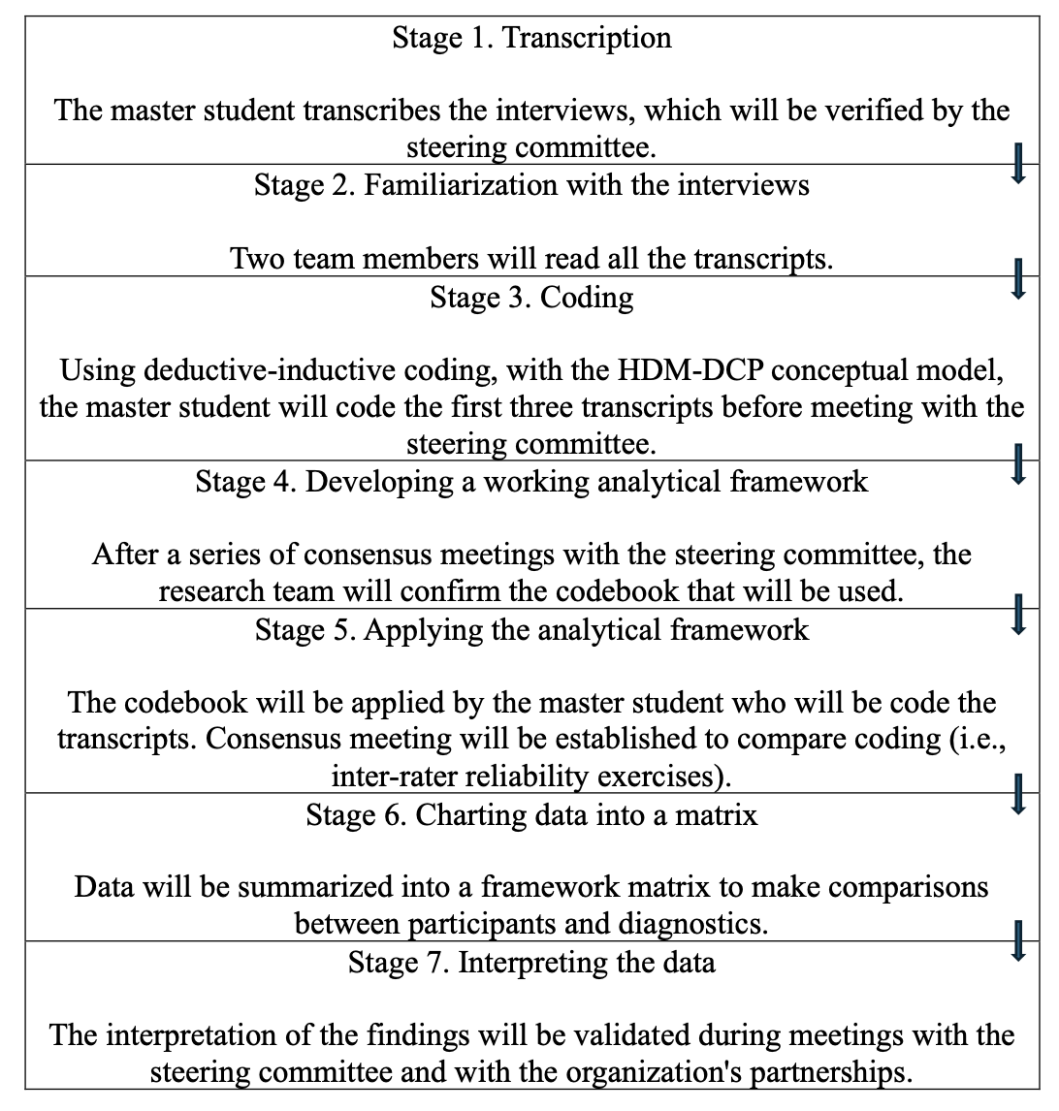
Application of the seven stages of the Framework method. HDM-DCP: Human Development Model–Disability Creation Process.

This study aims to coconstruct knowledge with individuals who have long-term neurological disabilities. It is part of a larger participatory action research project financed by the Canadian Institutes of Health Research (grant number 202403-TLP495860, 2023-2026). Figure 1 presents the entire procedure of the study.

### Study Design

Given the lack of prior studies addressing these objectives, this descriptive qualitative research serves as an exploratory study, providing an initial overview of key elements that can inform and guide future research in this area. In alignment with best governance practices in partnership-based research [35-37], a steering committee will be established. It will be composed of individuals aging with disabilities (n=3), representative of the sample size and aged between 50 and 65 years, community workers working with these populations (n=2), a research professional (n=1), the principal investigator (n=1), and a graduate student (n=1). The individuals chosen to compose the steering committee will ensure a good representation of the diagnoses of interest and different stakeholders. The partners have been longstanding collaborators with the research committee. This committee upholds and fosters best practices in partnership-based research, both of which are core values for the team [35-37]. The graduate student’s role within the committee will be to coordinate research activities throughout the entire process. The relevance of this study will be discussed and validated with the individuals aging with disabilities, and the community workers involved in the steering committee. For this qualitative research, the steering committee will also be involved in the review of data collection tools and the knowledge transfer plan [38].

The results of this project will be reported according to the COREQ (Consolidated Criteria for Reporting Qualitative Research) (see Multimedia Appendix 1) [39]. This qualitative research tool provides a 32-item checklist to ensure that all important aspects are reported. The COREQ checklist includes three domains guiding qualitative research reports (eg, research team and reflexivity, study design, analysis, and findings). The epistemological stance of the research team is based on social constructivism [28]. By adopting a constructivist perspective, the team is able to develop knowledge about aging and the social participation of individuals living with a TBI, MS, or a SCI, acknowledging that this understanding emerges from the lived experiences of those affected [28].

### Recruitment

The target population of this project consists of individuals aged 50 years and older, with a long-term neurological disability for more than 10 years. The choice of a duration since diagnosis will allow us to explore the aging experiences of individuals who have been adapting for a significant number of years and are no longer receiving rehabilitation services. Exclusion criteria include cognitive impairment causing an inability to consent freely and enlighten to the participation in the study. Three populations will be initially targeted: individuals aging with a TBI, an MS, or a SCI. The recruitment of 24 participants aged with one of the mentioned conditions (ie, TBI, SCI, or MS) will be divided equally among the three health conditions (n=8 per condition). Participants must meet the inclusion criteria that are presented in Textbox 1. For this exploratory study, data saturation will be assessed throughout data collection [40]. To ensure data saturation, we will use an iterative approach to data collection and analysis, continuously reviewing and refining our themes as new interviews are conducted [40]. In addition, we will use member checking, where preliminary findings will be shared with participants for validation and to identify any gaps in our understanding, thus enhancing the comprehensiveness of our data and ensuring that all relevant themes are captured. More participants could be recruited if data saturation is not reached with this sample size [41,42].

**Criteria for recruitment.**

**Inclusion criteria**

- 50 years old and older
- Diagnosed with traumatic brain injury (TBI), multiple sclerosis, or spinal cord injury for at least 10 years
- Moderate or severe TBI
- Spinal cord injury and multiple sclerosis, regardless of the type of diagnosis
- Ability to communicate in French or English

**Exclusion criteria**

- Cognitive impairment causing an inability to give consent
- Mild TBI

Recruitment will be done through purposive sampling to promote diversity in the profiles of recruited individuals (ie, sex, gender, diagnosis, time since diagnosis) [43]. Participant recruitment will be carried out in collaboration with research partnerships that have been established with four organizations in the province of Quebec (ie, Adaptavie, Servio, Multiple Sclerosis Canada, and Moelle épinière et motricité Québec). Each of these organizations provides support services and activities for individuals living with neurological disorders. Adaptavie’s mission is to provide individuals with functional limitations access to a diverse range of adapted physical, recreational, and social activities that promote inclusion, well-being, and active living. Moelle épinière et motricité Québec, formerly known as the Quebec Paraplegic Association and the Foundation for Spinal Cord Research, is a nonprofit organization dedicated to promoting self-sufficiency, independence, and quality of life for people with disabilities. Servio is also a nonprofit organization dedicated to individuals living with a traumatic brain injury. The eligibility criteria and interview procedures will be shared and discussed with our community partners. This collaboration aims to ensure a mutual understanding of the study objectives and to facilitate the identification of potential participants in an ethical and respectful manner. Our partners, with their expertise and in-depth knowledge of the community, will play a crucial role in the appropriate recruitment of interested individuals for our study. A snowball sampling strategy could be used if needed as a complementary approach [44]. Also, the sample of 24 people aging with a long-standing disability of neurological origin, MS (n=8), TBI (n=8), and SCI (n=8) will allow us to identify the major initial themes common or distinct between the participants in terms of their experiences of social participation and aging with a disability. The research team aims to describe in a rich and detailed manner the representations of the MS, TBI, and SCI clientele rather than only superficially reporting the representations of several distinct clienteles. Thus, the selection of participants does not aim to reflect the heterogeneity of the clientele. As a reminder, this is an exploratory project that will lay the foundations for the codevelopment of a health promotion program to address the challenges of social participation of populations aging with a neurological disorder. Furthermore, the choice to focus on these 3 clienteles and the sample size (n=8 per condition) also explains the contrasts which characterize them. It is crucial to emphasize the exploratory nature of this research. Our goal is to document key elements and major themes, both common and distinct, among participants regarding their experiences of social participation and aging with a disability. Rather than drawing stark contrasts, this approach will allow us to identify elements associated with aging with disabilities that transcend the specific nature of the impairment and are thus common across these populations. Our methodology seeks to establish a holistic understanding of the experiences of aging with a disability while acknowledging the particularities of each condition studied. By doing so, we will gain a better understanding of the shared challenges and unique experiences of each group.

### Data Collection

Data collection methods included obtaining consent from the participants, the conduct of the semistructured interview, and completing a sociodemographic questionnaire. As part of the development of the interview guide, the steering committee will be invited to comment on the relevance and clarity of the questions (see Multimedia Appendix 2 for the actual version of the guide). The interview guide and the sociodemographic questionnaire will be pretested with individuals representing each target population (n=3). Following the review of data collection tools, feedback will be integrated and validated by the steering committee members. Participants will be asked to complete a sociodemographic questionnaire (ie, sex, gender, age, ethnocultural origin, socioeconomic status, educational level, housing situation, marital status, diagnosis, rehabilitation history, and impacts on daily life). The interview will last between 60 and 90 minutes and will be conducted by the master’s student, who is a part-time community worker with individuals living with neurological disabilities for almost 2 years. To safeguard participants’ confidentiality and privacy, we have implemented a data management plan. All audio recordings and transcripts will be stored on a secure university server. Following data collection, personal identifiers will be promptly removed and replaced with unique codes, with the linking key accessible only to the principal investigator. Access to the deidentified data will be strictly limited to authorized research team members directly involved in data analysis [45]. Interviews will be audio recorded with a voice recorder and securely stored at The Interdisciplinary Research Center on Rehabilitation and Social Integration (Centre interdisciplinaire de recherche en réadaptation et intégration sociale; Cirris) and confidentially managed [45].

To maximize inclusion for participants with communication, physical, or memory challenges, strategies for full participation, such as flexible interview methods, will be used [46,47]. From the perspective of providing comfort during the interview, the sociodemographic questionnaire and a semistructured interview guide will be provided to each participant before the meeting. The research team is aware of the transportation challenges faced by participants. Thus, the team will demonstrate flexibility in the locations for the interviews, as well as in how they are conducted. And so, the interview will be conducted according to the participants’ preferences (eg, at home, in a research center room, or via video conference). The master’s student will initiate contact with selected participants via phone, maintaining sensitivity and attentiveness throughout the interaction. For interviews conducted via videoconference, any concerns about technology access will be addressed and resolved in advance [45]. This initial contact will serve as an opportunity to clarify questions about the project or interview process and establish optimal conditions for the interview [45]. The data collection process can be spread over as long a period as necessary to respect their capacity (ie, split the interview into 2 sessions or take a break).

### Data Analysis

Before the data analysis, the interviews will be transcribed verbatim. The first transcriptions will be validated by the research professional to ensure fidelity. Preliminary results will undergo consultation to support the utility and clarity of the themes emerging from the analyses. To ensure rigor, various validation steps will be conducted throughout the transcription and data analysis process (eg, double-blind validation and validation with quality assurance). Also, 12.5% of the transcripts will be validated by interrater reliability exercises by a neutral party. Before the qualitative analysis, the team will meet to ensure the unfolding of the Framework method [48]. The interest of this approach is that the Framework Method provides 7 steps to follow and produces highly structured outputs of summarized data [48]. Using this method will help structure the analysis method before beginning the qualitative analysis. The use of NVivo (version 15; Lumivero) software [49] for the analysis of qualitative data will make it possible to organize and structure the data from the interviews. Data analysis will be conducted using the mixed inductive-deductive thematic analysis method. The deductive part will rely on the Human Development Model–Disability Creation Process (HDM-DCP) [50]. This model presents three closely interconnected domains: lifestyle habits, personal factors, and environmental factors. Those domains mutually influence one another and collectively shape social participation. Our coding tree is derived from the detailed descriptors of the various components that constitute these domains, ensuring a comprehensive and theoretically grounded analysis of social participation. The use of this model allows understanding of the interaction between the individual, their environment, and their lifestyle habits [51]. The relevance of this model choice lies in the recognition of environmental factors alongside personal factors and lifestyle habits [51]. The environment plays an active role in the individual’s disability [51]. The inductive part of data analysis will allow themes to emerge from the verbatim. A descriptive analysis of sociodemographic data will be reported through summary descriptive statistics (ie, means, ranges, frequencies) to establish sociodemographic profiles of the encountered populations. Comparative analyses will be conducted to systematically examine differences (or similarities) based on variables such as sex, gender, or diagnosis. As mentioned, these populations are likely to offer a contrast between aging with a neurological disability. The results will establish sociodemographic profiles of the encountered populations.

### Ethical Considerations

This project has been approved by the ethics committee of the Centre intégré universitaire en santé et services sociaux de la Capitale Nationale (CIUSSS-CN; number 2024-2971). A monetary compensation of CAD $40 (US $29) was offered to each participant. Before the interview, each participant will be informed that their confidentiality will be protected and that they could choose not to answer any questions they find uncomfortable. Also, they will be free to withdraw from the research whenever they want. The interviewer will be attentive to signs of discomfort and will adjust as needed based on signs of discomfort. The purpose of the project and the required involvement will be briefly explained during the initial contact, and the informed consent form will be sent to each participant before the interview. Informed consent will be obtained before the interview by reading and signing the consent form. Thereby, every modification of the data tools will be approved by the ethics committee at any stage of modification, if applicable.

## Results

**Figure 2:**
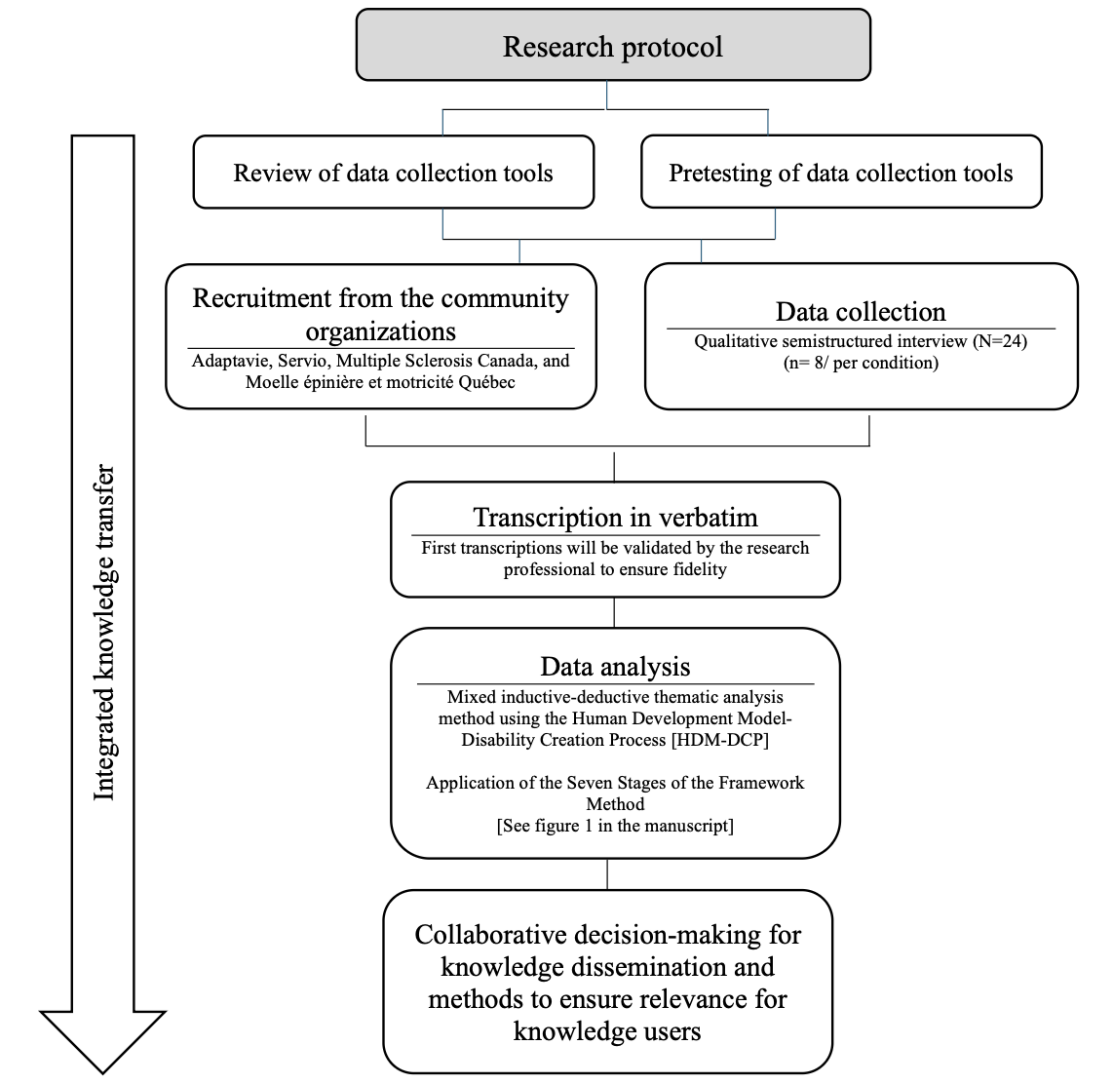
Study protocol chart.

The study opened to recruitment and data collection in April 2024. By December 2024, 24 participants have been interviewed. Following the 7 steps of the Framework method, the Application of the Seven Stages of the Framework Method (see Figure 2) demonstrates the process of the research project [48]. Data analysis is anticipated to begin in January 2025 and will provide evidence-based knowledge on the diversity of social participation experiences of these populations. It will determine, if applicable, commonalities in social participation and active aging, and which elements are specific to each population. We expected that there would be similarities between the SCI and MS populations according to their physical disability. This project will also shed light on different ways in which these populations wish to be supported in their participation, and the role of individual characteristics and environmental resources in shaping these experiences. The findings will prioritize the richness of social participation experiences over diagnostic differences, rather than focusing on the severity of impairments or variations within the same population (eg, type of MS, severity of TBI, or level of SCI). Findings and interpretations will be reported in line with guidance from the COREQ checklist [39]. We expected to begin the knowledge transfer plan during the spring and summer of 2025 [38]. Partner organizations will be consulted to determine the knowledge transfer methods to be used. The tools will be developed in alignment with the interests of the partners and their members [52]. Thus, the expected results are to be published in fall 2025.

## Discussion

### Principal Findings

This qualitative study is intended to explore the aging experiences of individuals with a long-term neurological disability and to describe the barriers and facilitators to achieving their social participation. This is the first study to explore the social participation experiences of 3 populations aging with a disability, as a TBI, an SCI, or an MS.

Methodologically, our study aims to adequately address considerations related to the principles of equity, diversity, and inclusion. To achieve this, the approaches will be adapted (eg, data collection strategies). Indeed, in the interest of inclusion, it is necessary to adjust to the participants’ abilities and offer flexibility in our approach to enable full participation. This inclusive stance is essential for the study, as it ensures greater scientific rigor by supporting the quality of the data collected.

Internationally, various health organizations, such as the World Health Organization (WHO), recognize disability as a significant factor in situations of inequality [53]. The WHO recognizes the need to develop coordinated actions to ensure that people with disabilities have equitable access to effective health care services [53]. Also, some organizations have developed action plans to improve community health, such as the Quebec Pride in Aging: Government Action Plan 2024-2029 [54] that recognizes the importance of social participation as a key determinant of seniors’ health. Social participation is positively associated with the health of individuals aging with disabilities [23], a reduced risk of mortality [24], and greater life satisfaction [23]. This study will undoubtedly establish important guidelines to promote the well-being of individuals aging with disabilities.

Social participation is a modifiable determinant of health. Various studies involving other populations living with a neurological condition addressed the barriers and facilitators to social participation of individuals aging with disabilities [11,55]. Others have also demonstrated the positive impact of enhancing social participation by modifying environments and providing assistive devices to promote it. An interdisciplinary team worked on the codevelopment of a toolkit that brings together strategies to promote the social participation of individuals aging with spina bifida, their families, and caregivers [56]. Also, some neurological disability (ie, SCI, cerebrovascular accident) can lead to important physical disability. Another research has demonstrated the positive impact of the use of a wheelchair to improve social participation [57]. The results of this study will enable researchers, professionals, and organizations to develop health services and care in alignment with the experiences explored in this research. Thus, by acting on social participation, these studies have shown positive outcomes for seniors living with disabilities.

### Limitations and Strengths

A key strength of this study is the large number of members in the organization partnerships who are involved in research and are guiding decision-making processes. Furthermore, the interviewer has clinical expertise with the populations under study in her role as a community worker. These conditions together form a strength in the implementation of this participatory research project.

Some limitations of this study are related to the health conditions regarding the neurological disability of the target populations. For example, some individuals with a TBI may be facing language disorders, such as aphasia or memory disorders. Thus, although some aging experiences with this clientele would be significant for the study, it would not be possible to transcribe the discussion or conduct an interview either.

In addition, while this study is exploratory and not intended to provide comprehensive knowledge, it will play a valuable role in informing future research and practice. The findings will contribute to a deeper understanding of the diverse experiences of aging and social participation within the population of interest, potentially paving the way for the development of best practices and evidence-based approaches in this field.

### Conclusion

This project will lay the groundwork for the codevelopment of health promotion programs that support the social participation of individuals aging with a TBI, MS, or SCI. Findings can guide not only health, community, and social service professionals but also disability rights organizations, caregivers, and individuals aging with disabilities themselves in how to move toward a more inclusive vision of aging and to dismantle systematic barriers to social participation in populations facing social and health inequities.

## Appendix

Multimedia Appendix 1 COREQ (Consolidated Criteria for Reporting Qualitative Research) checklist.

Multimedia Appendix 2 Semistructured interview guide.

## Abbreviations

Cirris: Centre interdisciplinaire de recherche en réadaptation et intégration sociale
CIUSSS-CN: Centre intégré universitaire en santé et services sociaux de la Capitale Nationale
COREQ: Consolidated Criteria for Reporting Qualitative Research
HDM-DCP: Human Development Model–Disability Creation Process
MS: multiple sclerosis
SCI: spinal cord injury
