# Supplementary material for: Social Participation When Aging With an Early-Onset Neurological Disability: Protocol for Descriptive Qualitative Research
Source: JMIR Res Protoc. 2025 Aug 6;14:e66963. doi: 10.2196/66963 (PMC12368468; doi:10.2196/66963)
Supplement: Multimedia Appendix 2 [file resprot_v14i1e66963_app2.docx]

**Appendix 1 - Semi-Structured Interview Guide**

1. Tell me about aging with [a traumatic brain injury, multiple sclerosis, or a spinal cord injury]. [Note: if there is no spontaneous response, proceed with question a)]
2. How is aging with [a traumatic brain injury, multiple sclerosis, or a spinal cord injury] different from aging in a person who does not have this condition?
3. Does aging with [a traumatic brain injury, multiple sclerosis, or a spinal cord injury] create specific needs? If so, which ones? [Note: Highlight the specific needs that are different from those of a person who does not live with a neurological disability]
4. How are you preparing to age with [a traumatic brain injury, multiple sclerosis, or a spinal cord injury]? [Note: Explore here modifications to the living environment, search for information or services, etc.]
5. How does aging with [a traumatic brain injury, multiple sclerosis, or a spinal cord injury] influence your involvement in the community? [Note: Explore the term community (i.e., social circle, frequented institutions, etc.)]
6. Are you able to do all the activities you want outside your home?
   1. If not, what would you like to do that you are not currently doing?
   2. When you do activities outside your home, with whom do you do these activities?
   3. What do you gain from doing these activities with these individuals?
7. What helps you maintain your participation in activities outside your home? [Note: Explore here the facilitators associated with the physical, social, and institutional environment. Also, explore elements related to the individual's characteristics, such as their strengths and abilities. Give examples of activities if needed].
   1. Do you think these facilitators will be different in the future or will evolve due to your aging? If so, can you tell me more? If not, why?

1. What limitations or obstacles do you encounter regarding your participation in activities? [Note: Explore here the obstacles associated with the physical, social, and institutional environment. Also, explore elements related to the individual's characteristics]
   1. How do these obstacles or limitations impact your aging?
   2. Do you think these obstacles will be different in the future or will evolve due to your aging? If so, can you tell me more? If not, why?
2. How can the community support your participation in activities that matter to you? [Note: Explore the term community (i.e., individuals who make up the social circle, individuals present in public places, various available services, transport, associations, and organizations, etc.)] [If the person talks about their participation in the activities of an association, you may ask the following question: How important is your membership in your organization in your aging?]
3. What would you need to improve your ability to do the activities you want outside your home? [Note: Explore here the ways to remove the obstacles to the participant's social participation mentioned in question 4 OR the ways to mobilize the strengths or facilitators of this same participation mentioned in question 3].
4. How can we support a fulfilling aging process for individuals who, like you, live with a disability? [Note: Explore if not addressed: What has been most helpful to you so far in helping you age in the way you want? What could make a difference in helping you age in a fulfilling way?]
   1. What would help you age in a fulfilling way? [Note: Explore the development of interventions, adaptation of places, creation or maintenance of a social support network, creation of opportunities for involvement in meaningful occupations.]
   2. What interventions are needed to help individuals like you who age with a long-term disability to do the activities and things that are important to them? [Note: Specify as needed; Interventions from healthcare professionals or services that help individuals with disabilities.]
5. Are there any elements we haven't discussed that you would have liked to share regarding your experience of aging with a disability? [Note: Explore the person's projection into the future, their achievements, etc.]
6. In your opinion, what should we take away from your experience of aging with [the diagnosis]?
